# Supplementary material for: Sub-millivolt voltage imaging reveals gap junction-mediated bioelectric contact inhibition
Source: Nat Commun. 2026 Aug 15;17:8414. doi: 10.1038/s41467-026-76758-z (PMC13477479; doi:10.1038/s41467-026-76758-z)
Supplement: Supplementary file 2 [file 41467_2026_76758_MOESM2_ESM.pdf]

Description of Additional Supplementary Information

File Name: Supplementary Data 1

Description: Sequence information for the expression vectors.

File Name: Supplementary Data 2

Description: Summary of *p*-values from the statistical tests performed.

File Name: Supplementary Data 3

Description: Gating strategy for FACS experiments.

File Name: Supplementary Movie 1

Description: Representative video of confluent Cx43/45 KO HEK293T cell lines with stable expression of rEstus2s, rEstus2s-T2A-Cx43, rEstus2s-T2A-ANO1, or rEstus2s-T2A-KCa3.1.

File Name: Supplementary Movie 2

Description: Representative video of confluent HEK293T, HaCaT, MCF-7, PANC-1, and A375 cell lines stably expressing rEstus2s.
