# Supplementary material for: Sub-millivolt voltage imaging reveals gap junction-mediated bioelectric contact inhibition
Source: Nat Commun. 2026 Aug 15;17:8414. doi: 10.1038/s41467-026-76758-z (PMC13477479; doi:10.1038/s41467-026-76758-z)
Supplement: Supplementary file 3 [file 41467_2026_76758_MOESM5_ESM.pdf]

BD FACSDiva 9.0.1

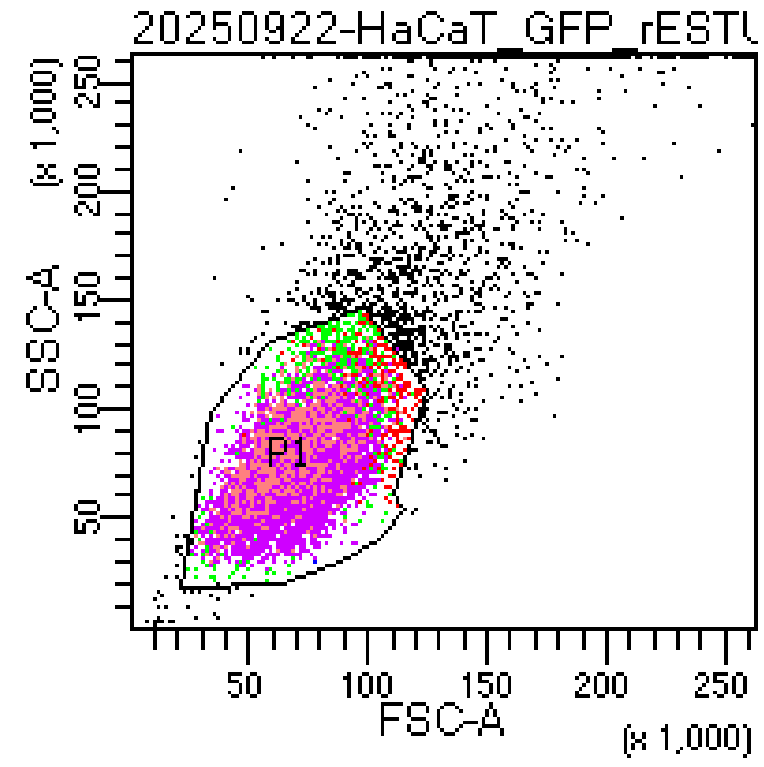

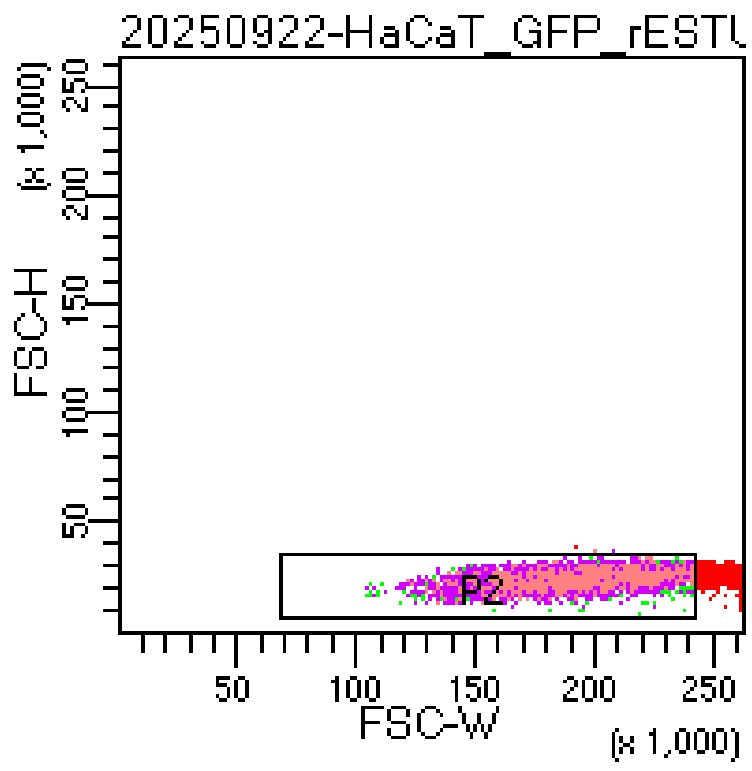

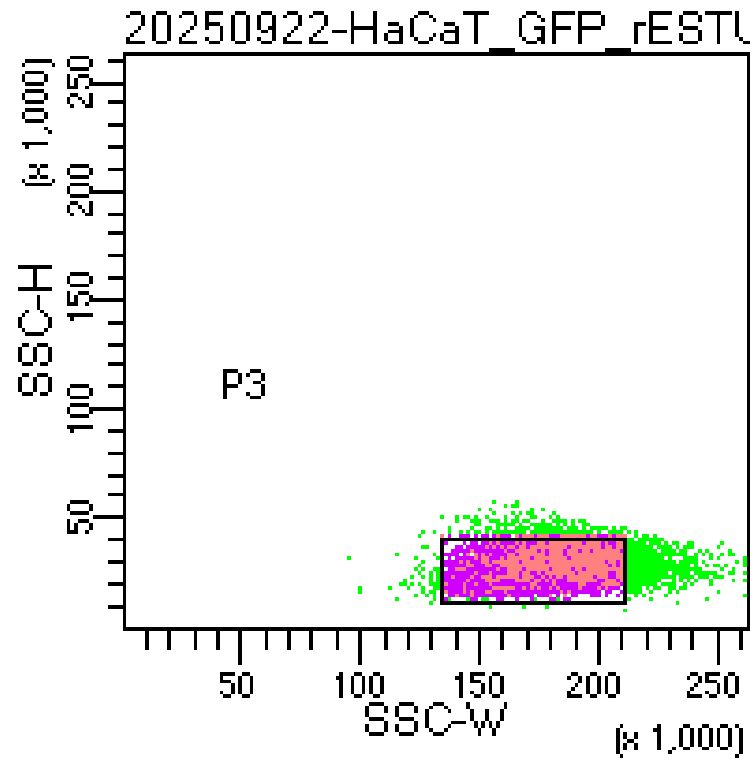

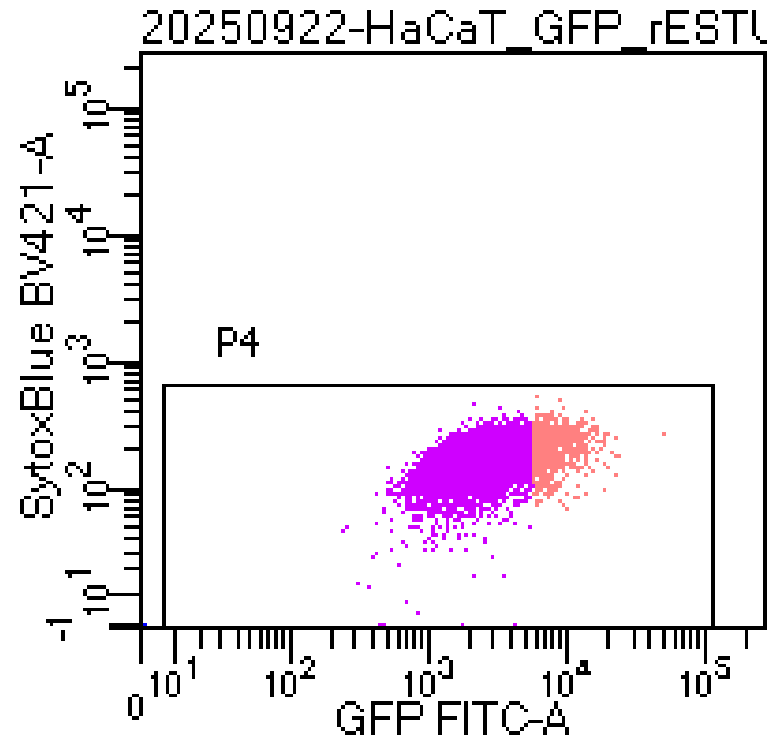

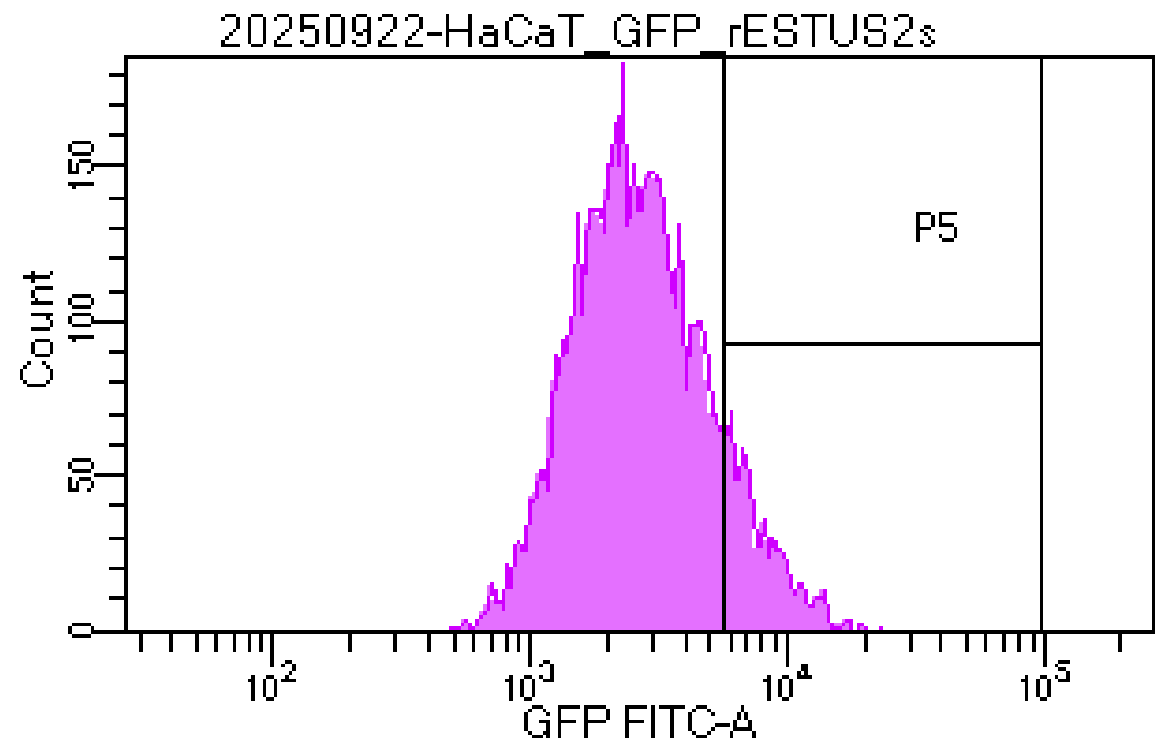

Tube: HaCaT_GFP_rESTUS2s

Population	#Events	%Parent	%Total
All Events	10,000	####	100.0
P1	8,347	83.5	83.5
P2	7,659	91.8	76.6
P3	6,532	85.3	65.3
P4	6,531	100.0	65.3
P5	799	12.2	8.0

BD FACSDiva 9.0.1

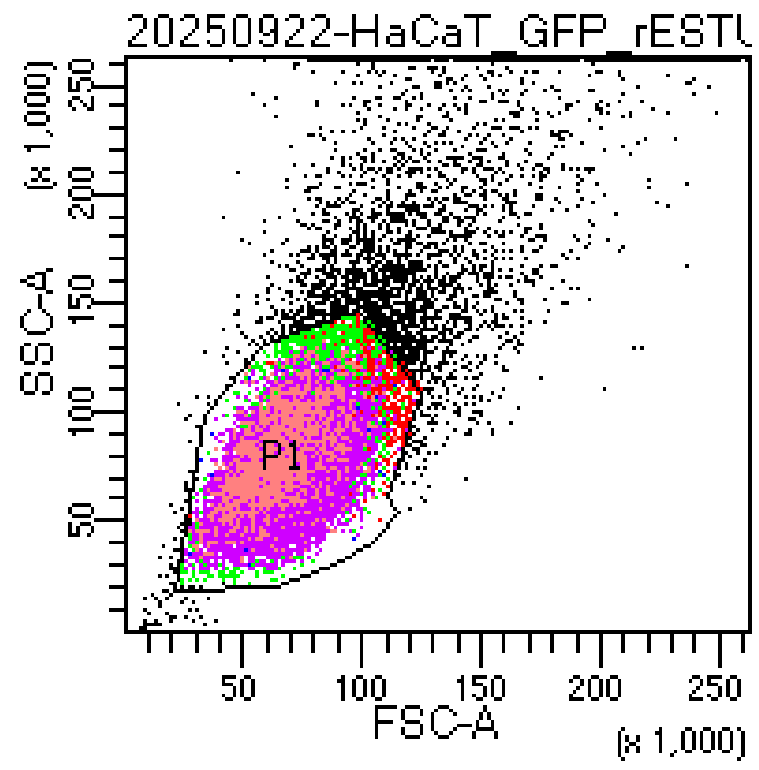

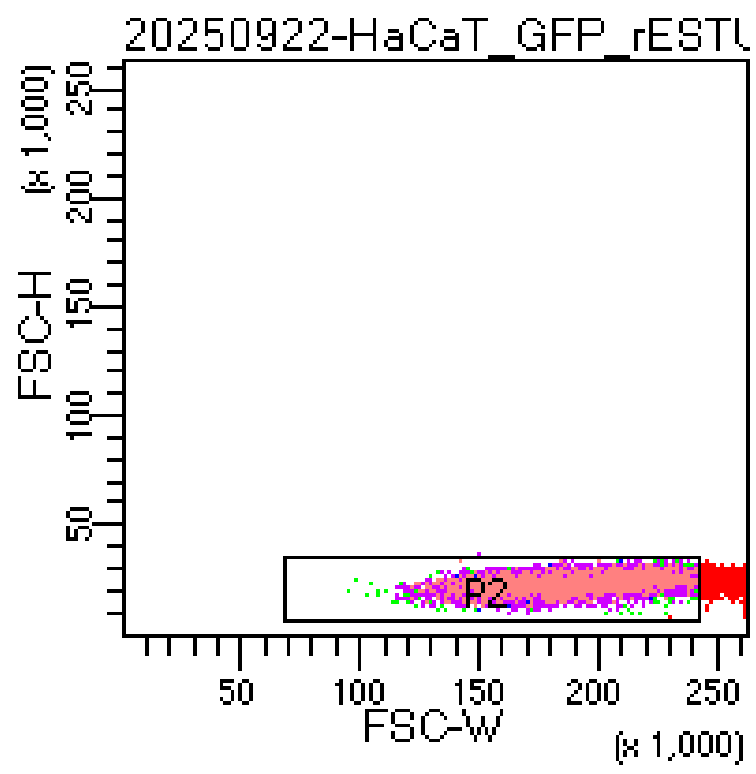

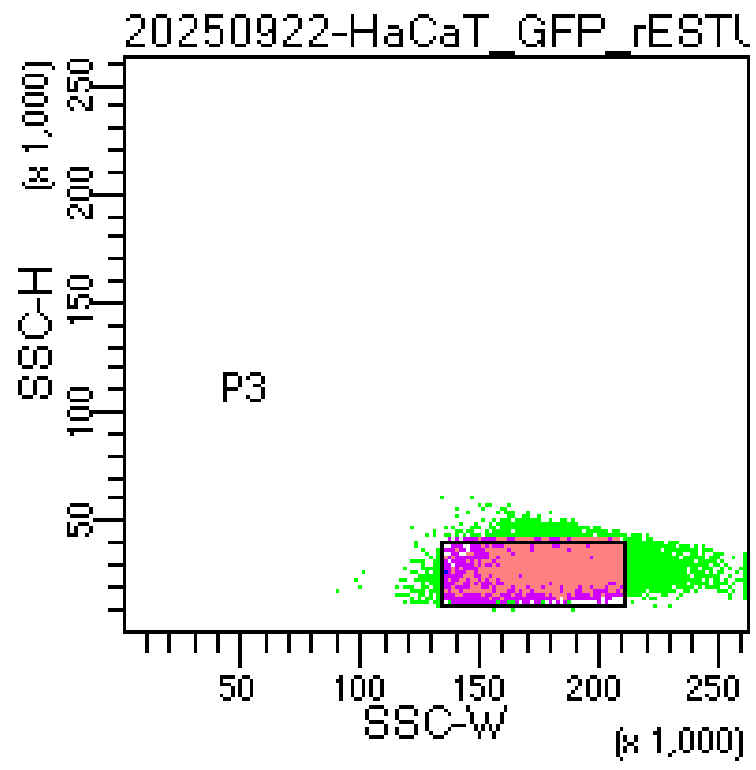

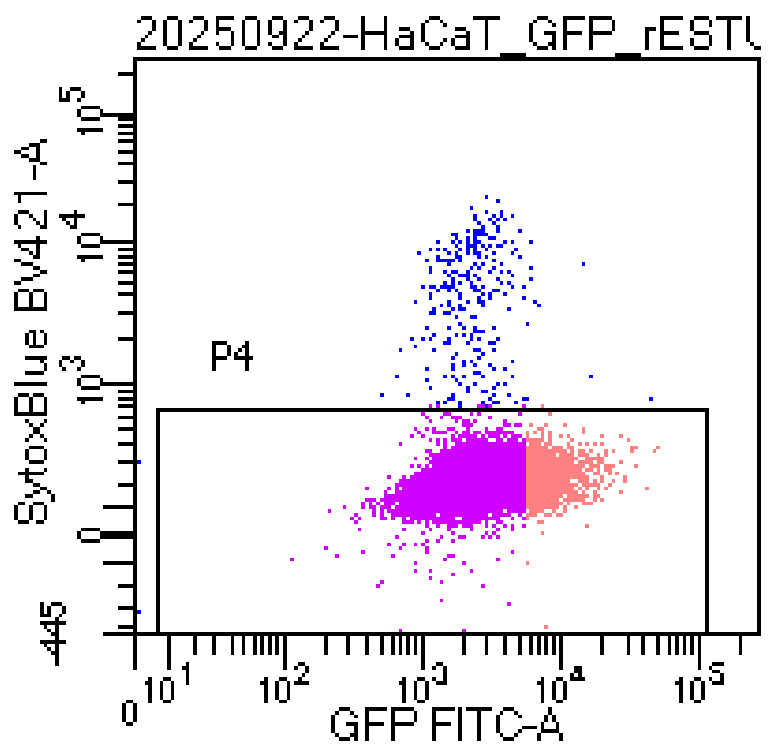

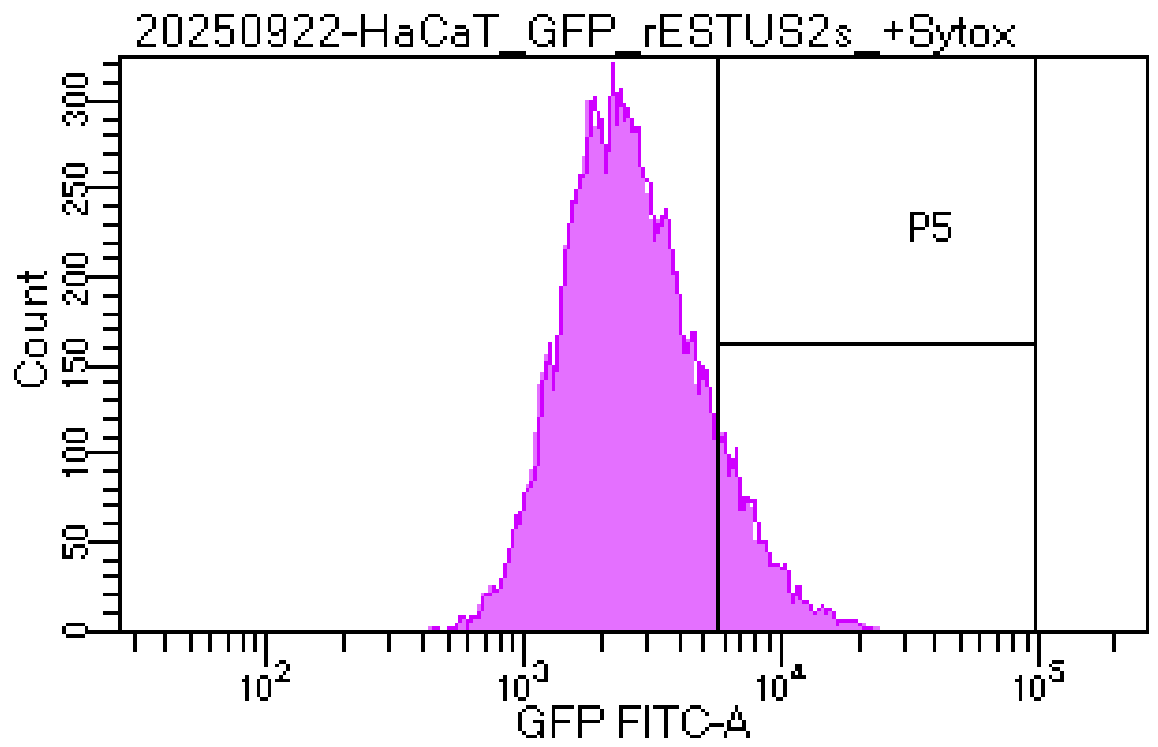

Tube: HaCaT_GFP_rESTUS2s_+Sytox

Population	#Events	%Parent	%Total
All Events	20,000	####	100.0
P1	16,763	83.8	83.8
P2	15,000	89.5	75.0
P3	12,683	84.6	63.4
P4	12,382	97.6	61.9
P5	1,451	11.7	7.3

BD FACSDiva 9.0.1

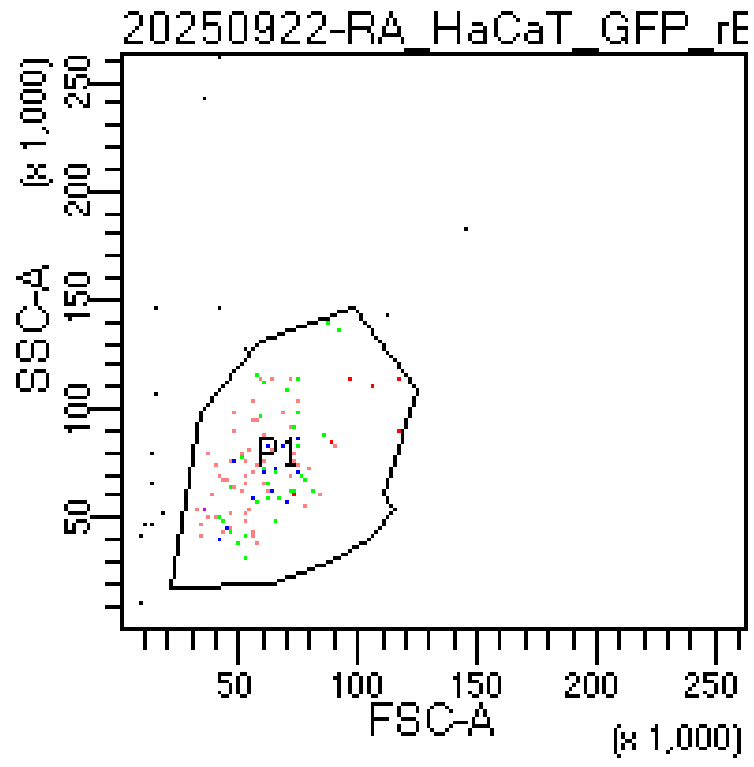

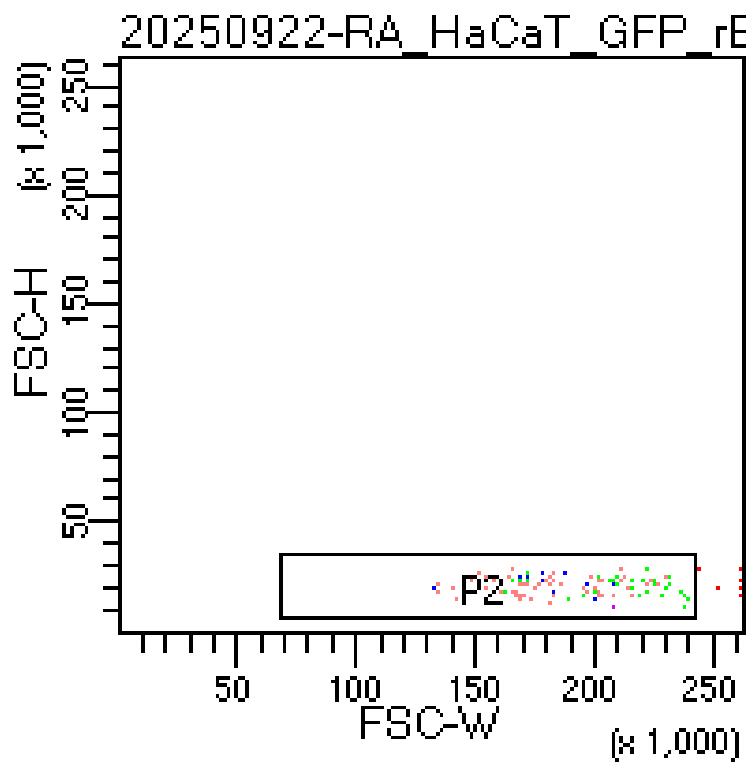

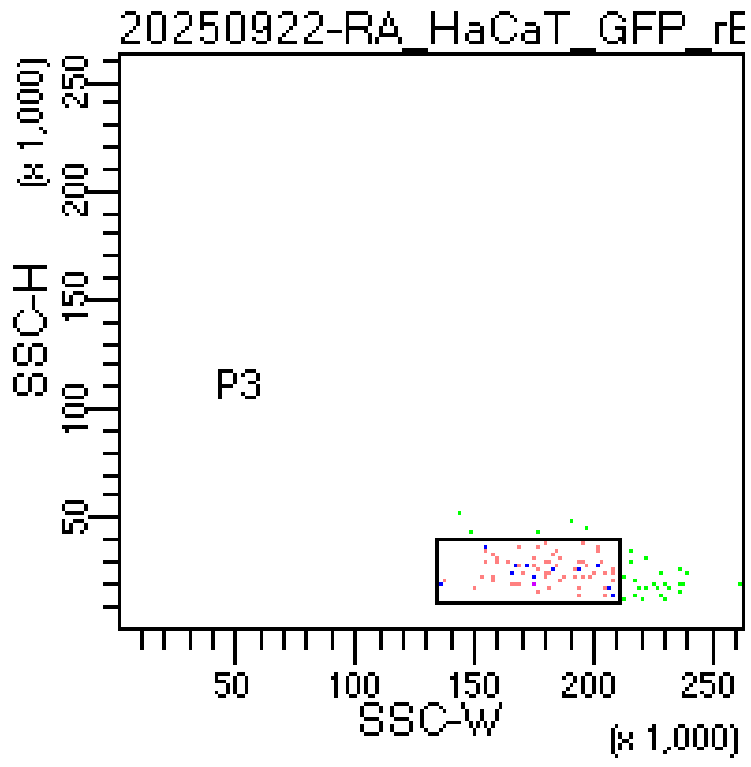

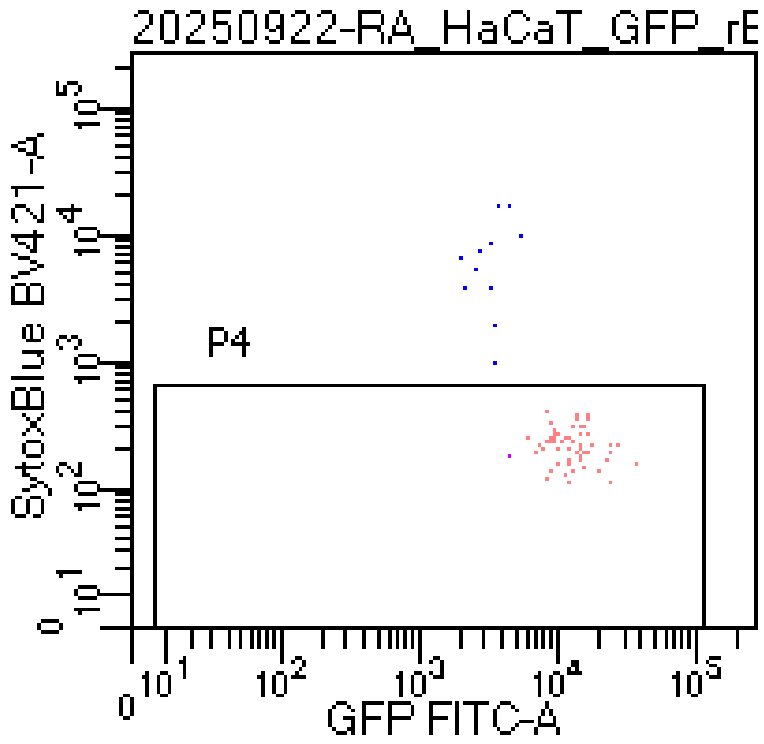

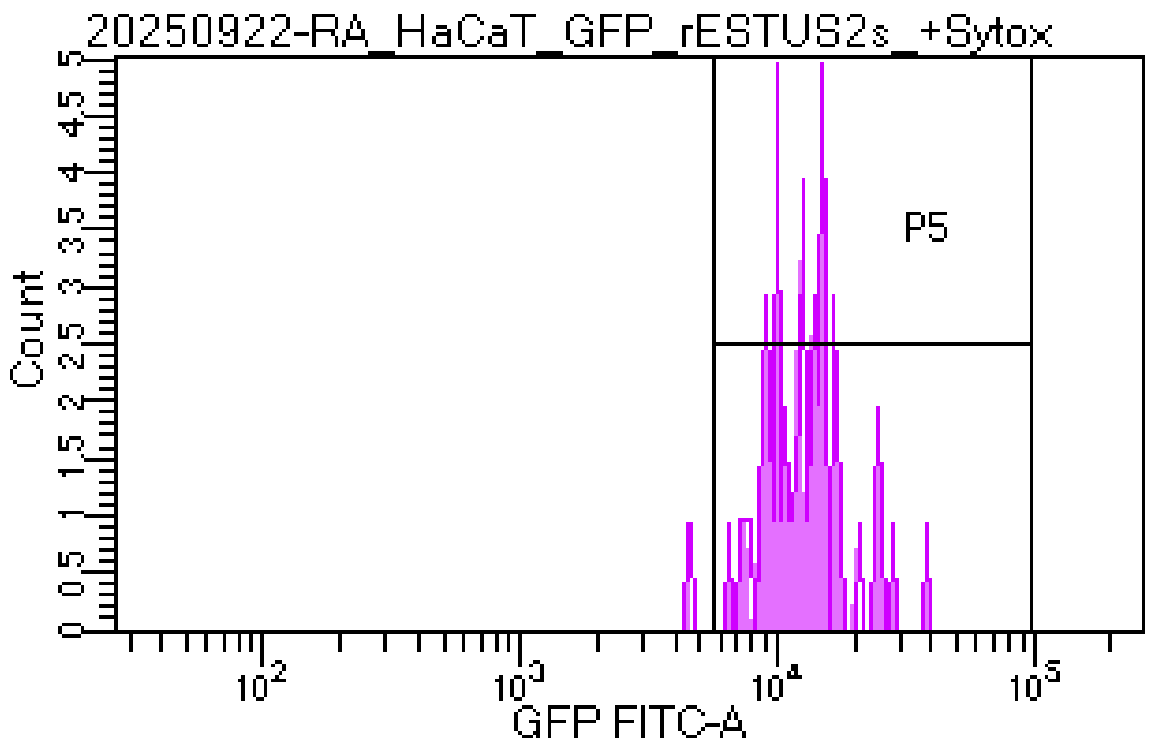

Tube: RA_HaCaT_GFP_rESTUS2s_+Sytox

Population	#Events	%Parent	%Total
All Events	125	####	100.0
P1	109	87.2	87.2
P2	102	93.6	81.6
P3	71	69.6	56.8
P4	59	83.1	47.2
P5	58	98.3	46.4
