# Supplementary material for: Sub-millivolt voltage imaging reveals gap junction-mediated bioelectric contact inhibition
Source: Nat Commun. 2026 Aug 15;17:8414. doi: 10.1038/s41467-026-76758-z (PMC13477479; doi:10.1038/s41467-026-76758-z)
Supplement: Supplementary file 4 [file 41467_2026_76758_MOESM8_ESM.pdf]

nature portfolio

Corresponding author(s): Philipp Rühl

Last updated by author(s): Jul 24, 2026

Reporting Summary

Nature Portfolio wishes to improve the reproducibility of the work that we publish. This form provides structure for consistency and transparency in reporting. For further information on Nature Portfolio policies, see our Editorial Policies and the Editorial Policy Checklist.

Statistics

For all statistical analyses, confirm that the following items are present in the figure legend, table legend, main text, or Methods section.

n/a	Confirmed	
☐	☒	The exact sample size (*n*) for each experimental group/condition, given as a discrete number and unit of measurement
☐	☒	A statement on whether measurements were taken from distinct samples or whether the same sample was measured repeatedly
☐	☒	The statistical test(s) used AND whether they are one- or two-sided<br>*Only common tests should be described solely by name; describe more complex techniques in the Methods section.*
☐	☒	A description of all covariates tested
☐	☒	A description of any assumptions or corrections, such as tests of normality and adjustment for multiple comparisons
☐	☒	A full description of the statistical parameters including central tendency (e.g. means) or other basic estimates (e.g. regression coefficient) AND variation (e.g. standard deviation) or associated estimates of uncertainty (e.g. confidence intervals)
☐	☒	For null hypothesis testing, the test statistic (e.g. *F*, *t*, *r*) with confidence intervals, effect sizes, degrees of freedom and *P* value noted<br>*Give P values as exact values whenever suitable.*
☒	☐	For Bayesian analysis, information on the choice of priors and Markov chain Monte Carlo settings
☒	☐	For hierarchical and complex designs, identification of the appropriate level for tests and full reporting of outcomes
☐	☒	Estimates of effect sizes (e.g. Cohen's *d*, Pearson's *r*), indicating how they were calculated

Our web collection on statistics for biologists contains articles on many of the points above.

Software and code

Policy information about availability of computer code

Data collection	The software tools and analysis procedures used are decribed in detail in the manuscript.
Data analysis	Raw image data were processed in Fiji (ImageJ). All subsequent signal processing, mathematical modeling, and statistical analyses were performed using Igor Pro 9 (WaveMetrics). Detailed procedures are provided in the Methods section.

For manuscripts utilizing custom algorithms or software that are central to the research but not yet described in published literature, software must be made available to editors and reviewers. We strongly encourage code deposition in a community repository (e.g. GitHub). See the Nature Portfolio guidelines for submitting code & software for further information.

Data

Policy information about availability of data

All manuscripts must include a data availability statement. This statement should provide the following information, where applicable:

- Accession codes, unique identifiers, or web links for publicly available datasets
- A description of any restrictions on data availability
- For clinical datasets or third party data, please ensure that the statement adheres to our policy

All data are available in the main text or the supplementary materials. The source data and raw data traces underlying all figures are provided in a Source Data file. Sequence information is provided in Supplementary Data 1. The rEstus2s expression vector is available from Addgene (250991). Due to large file sizes, the raw imaging data are available on request from the corresponding author. There are no restrictions on access, and requests will be processed within 4 weeks. The previously reported GFP protein structure used in this work is available via PDB ID 2Y0G [https://doi.org/10.2210/pdb2Y0G/pdb].

Research involving human participants, their data, or biological material

Policy information about studies with human participants or human data. See also policy information about sex, gender (identity/presentation), and sexual orientation and race, ethnicity and racism.

Reporting on sex and gender	-
Reporting on race, ethnicity, or other socially relevant groupings	-
Population characteristics	-
Recruitment	-
Ethics oversight	-

Note that full information on the approval of the study protocol must also be provided in the manuscript.

Field-specific reporting

Please select the one below that is the best fit for your research. If you are not sure, read the appropriate sections before making your selection.

[x] Life sciences [ ] Behavioural & social sciences [ ] Ecological, evolutionary & environmental sciences

For a reference copy of the document with all sections, see nature.com/documents/nr-reporting-summary-flat.pdf

Life sciences study design

All studies must disclose on these points even when the disclosure is negative.

Sample size	For imaging and patch-clamp experiments, sample sizes (n) are reported as the number of individual cells analyzed.
Data exclusions	No data were excluded from the analysis.
Replication	Key findings regarding Vm dynamics and bioelectric contact inhibition were robustly replicated across multiple independent cell lines as well as various experimental conditions. All attempts at replication were successful. Core biophysical principles were cross-validated using two different genetically encoded voltage indicators (rEstus and rEstus2s). For specific experimental datasets, data were compiled from 3 to 6 independent culture dishes.
Randomization	Randomization was not applicable to this study, as experimental groups were defined by specific cell types, genetic modifications (e.g., CRISPR-Cas9 knockouts), or the expression of specific ion channels and sensors.
Blinding	Blinding was not performed during data acquisition, as the investigators required knowledge of the specific cell lines and constructs to perform targeted patch-clamp and imaging protocols.

Reporting for specific materials, systems and methods

We require information from authors about some types of materials, experimental systems and methods used in many studies. Here, indicate whether each material, system or method listed is relevant to your study. If you are not sure if a list item applies to your research, read the appropriate section before selecting a response.

Materials & experimental systems

n/a	Involved in the study	
[ ]	[x]	Antibodies
[ ]	[x]	Eukaryotic cell lines
[x]	[ ]	Palaeontology and archaeology
[x]	[ ]	Animals and other organisms
[x]	[ ]	Clinical data
[x]	[ ]	Dual use research of concern
[x]	[ ]	Plants

Methods

n/a	Involved in the study	
[x]	[ ]	ChIP-seq
[ ]	[x]	Flow cytometry
[x]	[ ]	MRI-based neuroimaging

Antibodies

Antibodies used	Primary: Mouse monoclonal anti-Cx43 (sc-271837, 1:1000), anti-Cx45 (sc-374354, 1:1000), and anti-Vinculin (sc-73614, 1:2000) from Santa Cruz Biotechnology. Secondary: Peroxidase-conjugated goat anti-mouse (SeraCare, #5220-0341, 1:10000).

Validation	The anti-Cx43 (sc-271837) and anti-Cx45 (sc-374354) antibodies were validated by the complete absence of their respective protein bands in HEK293T Cx43 KO and Cx43/45 KO cell lines compared to wild-type controls. All primary antibodies, including anti-Vinculin (sc-73614), are commercially available and have been previously validated by the manufacturer (Santa Cruz Biotechnology) for use in Western blotting for human samples.

Eukaryotic cell lines

Policy information about cell lines and Sex and Gender in Research

Cell line source(s)	Lines used: HEK293T, MCF-7, A375, Panc-1, and HaCaT. Source: CAMR (HEK293T), ECACC (MCF-7 #86012803) ATCC (A375 #CRL-1619, Panc-1 #CRL-1469), and Cytion (HaCaT # 300493).
Authentication	Cell lines were authenticated by the supplier.
Mycoplasma contamination	All cell lines were confirmed to be negative for Mycoplasma contamination.
Commonly misidentified lines (See ICLAC register)	-

Plants

Seed stocks	-
Novel plant genotypes	-
Authentication	-

Flow Cytometry

Plots

Confirm that:

- [x] The axis labels state the marker and fluorochrome used (e.g. CD4-FITC).
- [x] The axis scales are clearly visible. Include numbers along axes only for bottom left plot of group (a 'group' is an analysis of identical markers).
- [x] All plots are contour plots with outliers or pseudocolor plots.
- [x] A numerical value for number of cells or percentage (with statistics) is provided.

Methodology

Sample preparation	Cells were harvested from confluent T75 culture flasks and sorted by fluorescence-activated cell sorting (FACS).
Instrument	Cell sorting was performed on a BD FACSAria™ IIIu (BD Biosciences, Franklin Lakes, NJ, USA) equipped with four lasers (V4, B2, YG4, R3) and a 100 µm nozzle.
Software	FACS data was analyzed with BD FACSDiva™ software version 9.0.1.
Cell population abundance	Cell lines were sorted to retain only the top 10% brightest cells based on GFP/FITC fluorescence intensity.
Gating strategy	Cell sorting was performed using a sequential gating strategy. First, the main cell population was identified on a forward scatter area (FSC-A) versus side scatter area (SSC-A) dot plot (P1), excluding debris and non-cellular events based on cell size and granularity. Doublets were subsequently excluded using FSC-width (FSC-W) versus FSC-height (FSC-H) (P2), followed by SSC-width (SSC-W) versus SSC-height (SSC-H) (P3). Dead cells were excluded using Sytox Blue staining on a BV421-A versus GFP/FITC-A dot plot (P4). Finally, the top 10% of GFP-positive cells, based on GFP/FITC fluorescence intensity, were selected on a GFP fluorescence histogram (P5). The P5 gate was used for cell sorting.

- [x] Tick this box to confirm that a figure exemplifying the gating strategy is provided in the Supplementary Information.
