# Supplementary material for: Sub-millivolt voltage imaging reveals gap junction-mediated bioelectric contact inhibition
Source: Nat Commun. 2026 Aug 15;17:8414. doi: 10.1038/s41467-026-76758-z (PMC13477479; doi:10.1038/s41467-026-76758-z)
Supplement: Supplementary file 5 [file 41467_2026_76758_MOESM9_ESM.pdf]

nature portfolio

Peer Review File

Sub-Millivolt Voltage Imaging Reveals Gap Junction-Mediated Bioelectric Contact Inhibition

Corresponding Author: Dr Philipp Rühl

This file contains all reviewer reports in order by version, followed by all author rebuttals in order by version.

Version 0:

Reviewer comments:

Reviewer #1

(Remarks to the Author)
The most significant results of the communication by Rhül et al. are:
i) the experimental demonstration of a genetically encoded voltage indicator that enables high-resolution of cell membrane potentials over a broad physiological range, which should be useful for understanding the electrophysiological dynamics of non-excitable cells; and
ii) the confirmation of gap junction-mediated bioelectric coupling in multicellular non-excitable networks.
This work could be of significance to the biophysical field because it provides a step further in the design of genetically encoded voltage indicators, both in terms of sensitivity and operation range, which constitutes a useful addition to the field. The authors have made a clear advance with respect to their previous work cited in the manuscript. Also, experimental results concerning the role of intercellular bioelectrical coupling are provided. Thus, the manuscript merits publication.
Some minor comments for the authors to consider:
(1) The theoretical scaling law relative to the number of cells in a network, which reflects a transition from stochastic single-cell behavior to collective electrical stability, constitutes a confirmation of previous biophysical models emphasizing the role of intercellular gap junction coupling in multicellular aggregates of non-excitable cells (Ref. 49 in the original manuscript but note also the full previous account in https://doi.org/10.1016/j.physrep.2022.12.003). Taken together, these results suggest how multicellularity could contribute to bioelectrical homeostasis, with potential implications in morphogenesis, regeneration, and cancer (Refs. 1 and 2 in the original manuscript).
(2) With reference to the conclusions and claims concerning point i) above, the authors may comment briefly on the possibility of altering normal cellular behavior by using genetically encoded voltage indicators. Despite this potential limitation, the interpretation and general conclusions drawn from the data analysis appear to be convincing. Also, the results obtained complement previous studies concerning low-frequency signals in cancer cells and can lead to new developments in basic science and applied diagnostics. The authors may also wish to check other bioelectrically-related work with voltage-sensitive dyes (https://doi.org/10.1038/s41567-019-0765-4) and microelectrode matrices (https://doi.org/10.1101/2024.03.16.585162).
(3) As to point ii) above, new and significant experimental evidence on the role of intercellular gap junctions in the bioelectric coupling of multicellular non-excitable networks is provided here. In this context, the authors may wish to check a recent manuscript (https://doi.org/10.1038/s41598-025-31696-6) that provides complementary but relevant evidence on the effects of gap junction on bioelectrical homeostasis.
(4) Emphasis is made on membrane potential fluctuations occurring at the sub-second time and sub-millivolt potential scales. An interesting addition for future studies would be to extend these scales to higher bioelectrical times and cell potentials, especially for long-term mapping of multicellular potentials in practical applications.
Moreover, it has been noted previously that changes in cell membrane potential could be related to proliferative processes. Again as future work, it could be interesting to compare the fast bioelectrical dynamics studied here with the slow changes observed in the cell polarization reported in previous studies, as mentioned in the manuscript.
(5) Please note that the observation that isolated cells and small clusters should be electrically volatile, together with the fact that a fast and localized current injection could act as an electrophysiological prompt that produces bioelectrical changes commensurate with the size of the neighboring cellular network, were theoretically predicted in https://pubs.acs.org/doi/10.1021/jp512900x
(6) Other questions of interest that may enhance further the potential impact of this and future works are:
(6.1) The results suggest that the membrane potentials fluctuations arise from spontaneous openings of ANO1 and KCa3.1 channels. This conclusion could be correct: Ca^{2+} electro-diffusion, for instance, should require times (at least) in the second

scale. Could the authors elaborate further this possibility by providing e.g. some approximate temporal estimates based on the channel conductances and the cell capacitance?
(6.2) Cell potential changes may involve both transient depolarizing and hyperpolarizing events. Also, spatio-temporal modules (subpopulations) with different cell polarizations are possible. In future studies, the authors are suggested to consider the long-term consequences of these facts, which could of high significance for morphogenesis and regeneration (Ref. 1 in the original manuscript).
In conclusion, we recommend publication of this interesting manuscript after the authors assess our minor remarks above, considering the suggested optional changes in the original manuscript.

Reviewer #2

(Remarks to the Author)
The lack of rigorous tools to estimate resting membrane potential is one of the largest challenges facing the field of bioelectricity, applied to numerous aspects of the life sciences. In this paper the authors develop rEstus2, a powerful iterative step forward that we think certainly warrants publication in Nature Communications. Using rEstus the authors expand on their previously published findings to demonstrate bioelectric contact inhibition (BCI) which reduces bioelectric volatility as collective size increases. They molecularly characterize BCI and attribute it to gap junctions, but find that BCI does not affect calcium dynamics. They then integrate these data into a descriptive model. This is a very nice paper, and there are only a couple of comments:

-The strongest alternative technology for optically estimating membrane potential is the PeT dye system designed by Evan Miller's group. Recent work using Fluorescent Lifetime Imaging (FLIM) has greatly improved the utility of PeT dyes for estimating resting membrane potential (refs 1-4 below), but the authors do not mention the PeT system in the current version of this manuscript. Miller's PeT system has recently been used to demonstrate long-distance propagation of bioelectric but not calcium dynamic signals (currently in preprint, #5 below), which has interesting implications for this paper that probably should be discussed. A table comparing published relevant values for PeT dyes with rEstus1 and 2 and ASAP3 (as well as any others the authors find helpful) could be helpful to properly contextualize rEstus2.

-rEstus2 is a powerful addition to the bioelectric toolkit, but its lack of ratiometry and lower brightness should be discussed in context of rEstus1. This is not a problem, and the sensitivity improvements of rEstus2 warrant publication in Nature Communications. But it might be good for readers to more clearly contextualizing rEstus2 as an alternative tool to rEstus1 instead of a clear 100% upgrade, to help readers decide on the best tool for their application.

-The authors effectively demonstrate the Bioelectric Contact Inhibition (BCI) phenomenon and convincing implicate Connexin activity in its function. Their model (Figure 6a-6b) that gap junctions facilitate a formation of a functional syncytium is not supported by the lack of synchrony in calcium dynamics. Their explanation for this discrepancy needs more support with referenced literature. Many gap junctions are voltage gated, so would it be possible for example that fast bioelectric signals are able to pass through gap junctions before calcium waves are able to propagate? In any case, the contradictory calcium data should be clarified as a knowledge gap that must be addressed in future work.

-The idea of cell couples as an intermediate between individual and confluent cultures is quite clever, and a useful datapoint, but their assertion starting line 2 that "If suppression of Vm fluctuations in confluent cultures were primarily driven by physical cell-cell contact and activation of membrane surface receptor pathways, a substantial reduction in Vm volatility would be expected upon formation of cell pairs." is questionable without extensive literature support. A confluent cell layer is highly likely to be very different from a single or couple of cells in many critical ways, and it would be good to contextualize their claim with that in mind.

-The authors discuss the interactions between membrane potential and Erk signaling document dynamic bioelectric signals regulated by collectivity. There is a growing body of literature that the Erk pathway temporally encodes information (see refs 6,7 below) and it would improve the impact of this manuscript were the authors to comment on the implications of the dynamic bioelectric signals they describe on the temporal dynamics of Erk signaling.

-In Figure 2H, could the difference in spectral density differences be attributed to capacitive current differences arising from the differential voltage sensitivity of the sensors?

We congratulate the authors on a very nice contribution to the field.

References cited:
1 Lazzari-Dean, J. R., Gest, A. M. M. & Miller, E. W. Optical estimation of absolute membrane potential using fluorescence lifetime imaging. eLife 8, e44522 (2019). https://doi.org:10.7554/eLife.44522
2 Lazzari-Dean, J. R. & Miller, E. W. Optical Estimation of Absolute Membrane Potential Using One- and Two-Photon Fluorescence Lifetime Imaging Microscopy. Bioelectricity 3, 197-203 (2021). https://doi.org:10.1089/bioe.2021.0007
3 Gest, A. M. M., Grenier, V. & Miller, E. W. Optical Estimation of Membrane Potential Values Using Fluorescence Lifetime Imaging Microscopy and Hybrid Chemical-Genetic Voltage Indicators. Bioelectricity 6, 34-41 (2024). https://doi.org:10.1089/bioe.2023.0027
4 Gest, A. M. M. et al. A red-emitting carborhodamine for monitoring and measuring membrane potential. Proc Natl Acad Sci U S A 121, e2315264121 (2024). https://doi.org:10.1073/pnas.2315264121

5 McMillen, P. & Levin, M. The neural crest as a bioelectric Rosetta Stone: translating the analog and digital bioelectric code with Fluorescent Lifetime Imaging (FLIM). . OSF Preprints (2025). https://doi.org:10.31219/osf.io/ncx84_v1
6 Purvis, J. E. & Lahav, G. Encoding and decoding cellular information through signaling dynamics. Cell 152, 945-956 (2013). https://doi.org:10.1016/j.cell.2013.02.005
7 Goglia, A. G. et al. A Live-Cell Screen for Altered Erk Dynamics Reveals Principles of Proliferative Control. Cell Syst 10, 240-253 e246 (2020). https://doi.org:10.1016/j.cels.2020.02.005

Reviewer #3

(Remarks to the Author)
I co-reviewed this manuscript with one of the reviewers who provided the listed reports. This is part of the Nature Communications initiative to facilitate training in peer review and to provide appropriate recognition for Early Career Researchers who co-review manuscripts.

Reviewer #4

(Remarks to the Author)
I co-reviewed this manuscript with one of the reviewers who provided the listed reports. This is part of the Nature Communications initiative to facilitate training in peer review and to provide appropriate recognition for Early Career Researchers who co-review manuscripts.

Version 1:

Reviewer comments:

Reviewer #1

(Remarks to the Author)

The authors have now addressed all my minor comments for improvement.

Thus, I should recommend the revised manuscript in its present form.

Reviewer #2

(Remarks to the Author)
I think the authors did a really nice job responding to all the comments, and the manuscript is much improved. I think it can be published now, I have no more concerns.

My co-reviewer said:

> My one comment is that I would like to see an addition to the paragraph at the end of their page 10 of the response document noting that ratiometry or FLIM are key in addressing artifacts stemming from cell shape change or differential sensor expression/staining.

Reviewer #3

(Remarks to the Author)
I co-reviewed this manuscript with one of the reviewers who provided the listed reports. This is part of the Nature Communications initiative to facilitate training in peer review and to provide appropriate recognition for Early Career Researchers who co-review manuscripts.

Reviewer #4

(Remarks to the Author)
I co-reviewed this manuscript with one of the reviewers who provided the listed reports. This is part of the Nature Communications initiative to facilitate training in peer review and to provide appropriate recognition for Early Career Researchers who co-review manuscripts.

Open Access This Peer Review File is licensed under a Creative Commons Attribution 4.0 International License, which permits use, sharing, adaptation, distribution and reproduction in any medium or format, as long as you give appropriate credit to the original author(s) and the source, provide a link to the Creative Commons license, and indicate if changes were made.
In cases where reviewers are anonymous, credit should be given to 'Anonymous Referee' and the source.
The images or other third party material in this Peer Review File are included in the article's Creative Commons license, unless indicated otherwise in a credit line to the material. If material is not included in the article's Creative Commons license and your intended use is not permitted by statutory regulation or exceeds the permitted use, you will need to obtain permission directly from the copyright holder.
To view a copy of this license, visit https://creativecommons.org/licenses/by/4.0/

REVIEWER COMMENTS

Changes to the text of the manuscript are highlighted in yellow

All figures and titles have been updated according to the *Nature Communications* formatting guidelines. The abstract has been revised according to the guidelines.

Figure 4 was missing indicators of statistical significance; indicators have now been added in the revised version.

Reviewer #1 (Remarks to the Author):

The most significant results of the communication by Rhül et al. are:
i) the experimental demonstration of a genetically encoded voltage indicator that enables high-resolution of cell membrane potentials over a broad physiological range, which should be useful for understanding the electrophysiological dynamics of non-excitable cells; and
ii) the confirmation of gap junction-mediated bioelectric coupling in multicellular non-excitable networks.
This work could be of significance to the biophysical field because it provides a step further in the design of genetically encoded voltage indicators, both in terms of sensitivity and operation range, which constitutes a useful addition to the field. The authors have made a clear advance with respect to their previous work cited in the manuscript. Also, experimental results concerning the role of intercellular bioelectrical coupling are provided. Thus, the manuscript merits publication.
Some minor comments for the authors to consider:

Response: Thank you for the careful reading of our manuscript and for highlighting its key contributions. We appreciate your recognition that this work advances our previous efforts and contributes to the broader biophysical understanding of non-excitable cellular networks. We also thank you for your recommendation for publication and for the constructive comments.

(1) The theoretical scaling law relative to the number of cells in a network, which reflects a transition from stochastic single-cell behavior to collective electrical stability, constitutes a confirmation of previous biophysical models emphasizing the role of intercellular gap junction coupling in multicellular aggregates of non-excitable cells (Ref. 49 in the original manuscript but note also the full previous account in https://doi.org/10.1016/j.physrep.2022.12.003). Taken together, these results suggest how multicellularity could contribute to bioelectrical homeostasis, with potential implications in morphogenesis, regeneration, and cancer (Refs. 1 and 2 in the original manuscript).

Response: We agree that the observed scaling behavior is consistent with previously proposed theoretical frameworks on intercellular coupling. We have revised the manuscript to explicitly acknowledge this prior work.

We now cite Cervera *et al.* (https://doi.org/10.1016/j.physrep.2022.12.003) and have added a clarifying statement in the text:

A similar community effect was previously described in theoretical models by Cervera *et al.*.

(2) With reference to the conclusions and claims concerning point i) above, the authors may comment briefly on the possibility of altering normal cellular behavior by using genetically encoded voltage indicators. Despite this potential limitation, the interpretation and general conclusions drawn from the data analysis appear to be convincing. Also, the results obtained complement previous studies concerning low-frequency signals in cancer cells and can lead to new developments in basic science and applied diagnostics.

Response: As with other voltage imaging approaches, GEVIs can in principle influence membrane properties, for example, through gating currents that may slightly affect membrane capacitance. However, similar or greater perturbations are also well documented for voltage-sensitive dyes, which additionally often introduce phototoxic effects. In our study, most measurements were performed in stably expressing cell lines, implying that cells tolerated indicator expression over extended periods. Moreover, expression levels in such systems are typically lower than in transient transfections, reducing the likelihood of significant physiological disruption. While we cannot fully exclude minor perturbations, we find no evidence suggesting detrimental or indicator-specific effects associated with rEstus2s.

The authors may also wish to check other bioelectrically-related work with voltage-sensitive dyes (https://doi.org/10.1038/s41567-019-0765-4) and microelectrode matrices (https://doi.org/10.1101/2024.03.16.585162).

Response: These works further support the relevance of both steady-state and dynamic bioelectrical signals in contexts such as pattern formation and cancer, and they complement our findings.

We have now included both references in the revised manuscript.

(3) As to point ii) above, new and significant experimental evidence on the role of intercellular gap junctions in the bioelectric coupling of multicellular non-excitable networks is provided here. In this context, the authors may wish to check a recent manuscript (https://doi.org/10.1038/s41598-025-31696-6) that provides complementary but relevant evidence on the effects of gap junction on bioelectrical homeostasis.

Response: Thank you for pointing out this interesting and relevant study. We agree that it provides complementary insight into the role of gap junctions in bioelectrical homeostasis. The results (e.g., Fig. 5) support the idea that cells may infer system size through the bioelectric state of their neighbors, which is conceptually aligned with our observations of collective electrical behavior. This raises an important open question as to whether such information is primarily encoded in steady-state (DC) signals, dynamic (AC) signals, or a combination of both, an aspect that will require further experimental investigation.

We have now included this reference in the revised manuscript.

We also included the following paragraph:

A fundamental open question is whether V_m is instructive and, if so, whether the information is predominantly encoded as sustained steady-state changes or as transient fluctuations. It is well established that signaling outcomes can be encoded in the temporal dynamics of pathways such as ERK.[71] For example, transient ERK activation promotes proliferation, whereas sustained ERK activation drives differentiation.[72] Furthermore, stochastic ERK activity pulses regulate proliferation rates in a cell density-dependent manner.[73] Thus, if ERK signaling is modulated by V_m, as suggested by previous studies,[6,7] its dynamics may likewise be shaped by the collective electrical properties of the multicellular network.

(4) Emphasis is made on membrane potential fluctuations occurring at the sub-second time and sub-millivolt potential scales. An interesting addition for future studies would be to extend these scales to higher bioelectrical times and cell potentials, especially for long-term mapping of multicellular potentials in practical applications.
Moreover, it has been noted previously that changes in cell membrane potential could be related to proliferative processes. Again as future work, it could be interesting to compare the fast bioelectrical dynamics studied here with the slow changes observed in the cell polarization reported in previous studies, as mentioned in the manuscript.

Response: Thank you for this suggestion. We agree that extending measurements to longer timescales and a larger voltage range is an important direction, particularly for understanding bioelectrical dynamics in more complex multicellular systems.

As network size increases, passive electrical properties such as RC filtering are expected to play a more prominent role, effectively acting as a low-pass filter and favoring slower potential fluctuations. This raises the intriguing possibility that large multicellular assemblies or tissues may exhibit emergent collective behaviors, such as preferred oscillatory modes, that constrain the range of observable bioelectrical dynamics. Consistent with this idea, we have observed slow-wave oscillations in sub-networks of PANC-1 cells (see Supplementary Video 2).

We also agree that linking fast bioelectrical fluctuations and slower changes in membrane potential associated with processes such as proliferation or differentiation is an important open question. Extending our approach to physiologically relevant systems, such as stem cell-derived organoids, will be particularly valuable in this context. In this regard, we have recently demonstrated the applicability of the original rEstus sensor in induced pluripotent stem cells (https://doi.org/10.1016/j.scr.2026.103972), which provides a foundation for future studies of long-term and large-scale bioelectrical pattern formation during development and tissue growth.

To reflect this, we have added the reference and included the following statement in the manuscript:

However, the functional role of electrical coupling and signaling in non-excitable cells remains incompletely understood and requires further investigation in physiologically relevant models, such as organoids derived from induced pluripotent stem cells.

(5) Please note that the observation that isolated cells and small clusters should be electrically volatile, together with the fact that a fast and localized current injection could act as an electrophysiological prompt that produces bioelectrical changes commensurate with the size of the neighboring cellular network, were theoretically predicted in https://pubs.acs.org/doi/10.1021/jp512900x

Response: Thank you for this important remark. We agree that this phenomenon is consistent with prior theoretical work.

We have now included the suggested reference in the manuscript:

(6) Other questions of interest that may enhance further the potential impact of this and future works are:
(6.1) The results suggest that the membrane potentials fluctuations arise from spontaneous openings of ANO1 and KCa3.1 channels. This conclusion could be correct: Ca2+ electro-diffusion, for instance, should require times (at least) in the second scale. Could the authors elaborate further this possibility by providing e.g. some approximate temporal estimates based on the channel conductances and the cell capacitance?

Response: Thank you for this insightful question. The observation that both depolarizing (ANO1-mediated) and hyperpolarizing (K_{Ca}3.1-mediated) events require intracellular Ca^{2+}, yet do not correlate with detectable global Ca^{2+} transients, strongly suggests that these fluctuations are driven by highly localized and spatially confined Ca^{2+} micro-/nanodomains rather than by cell-wide Ca^{2+} signals. This interpretation is further supported by the fact that both event types are abolished by BAPTA-AM, indicating a strict dependence on Ca^{2+} availability.

Interestingly, the durations of these events differ substantially: ANO1-mediated depolarizing events exhibit an average duration of approximately 0.58 s, whereas K_{Ca}3.1-mediated hyperpolarizing events persist for approximately 1.14 s. This difference may reflect distinct intrinsic gating kinetics of the two channel types. Alternatively, both channels could be activated by similar localized Ca^{2+} release events, with their temporal profiles being differentially shaped by membrane potential feedback (driving force change) or by channel-specific biophysical properties. A third possibility is that ANO1 and K_{Ca}3.1 are coupled to distinct classes of subcellular Ca^{2+} signals.

A quantitative estimate based on simple electrical considerations supports the idea that only a small number of channels are involved in these events. For traces with clearly resolved hyperpolarizing transients, we estimate characteristic time constants of approximately 200–400 ms for voltage excursions of ~-40 mV (to the K^+ reversal potential). Assuming a single-channel conductance of ~25 pS (K_{Ca}3.1) and a membrane capacitance of ~15 pF, the observed time constants correspond to an effective conductance change equivalent to approximately 2–3 simultaneously open channels under a simplified RC approximation. Because intact cells contain additional background conductance and non-ideal electrical properties, the actual number of participating channels may be somewhat higher, but likely remains well below the total channel population (≈1000) estimated from whole-cell recordings.

Together, these estimates support a model in which spontaneous, spatially restricted Ca^{2+} micro-/nanodomain activity gates small channel clusters.

(6.2) Cell potential changes may involve both transient depolarizing and hyperpolarizing events. Also, spatio-temporal modules (subpopulations) with different cell polarizations are possible. In future studies, the authors are suggested to consider the long-term consequences of these facts, which could of high significance for morphogenesis and regeneration (Ref. 1 in the original manuscript).

Response: Thank you for these insightful suggestions. We fully agree that transient depolarizing and hyperpolarizing events, as well as the emergence of spatiotemporal subpopulations with distinct polarization states, may have important long-term consequences for processes such as morphogenesis and regeneration.

Indeed, we have already observed evidence of such subsystems in certain cell types. In particular, within tumor-derived cell populations, gap-junction coupling is often heterogeneous, which may lead to partial electrical uncoupling and the formation of distinct bioelectrical sub-networks. For example, in PANC-1 cells we observed that some cells appear to be largely uncoupled from the surrounding network, whereas others form coupled sub-networks that exhibit slow oscillatory dynamics.

We agree that the long-term functional consequences of these dynamics remain poorly understood, especially with respect to how multicellular V_m states interact with other dynamic subcellular signaling systems. Because V_m is an emergent systems-level property, it is particularly well suited as a global readout of collective tissue state. An important next step will be to determine how these multicellular electrical dynamics relate to intracellular state transitions and downstream signaling programs. We are confident that the tools developed here will make addressing these fundamental questions technically feasible in future studies.

In conclusion, we recommend publication of this interesting manuscript after the authors assess our minor remarks above, considering the suggested optional changes in the original manuscript.

Response: We thank the reviewer for the careful evaluation of our manuscript and for the recommendation for publication. We appreciate the constructive and thoughtful comments, which have helped us to further improve the clarity and contextualization of our work. We have carefully addressed all minor remarks and incorporated the suggested revisions.

Reviewer #2 (Remarks to the Author):

The lack of rigorous tools to estimate resting membrane potential is one of the largest challenges facing the field of bioelectricity, applied to numerous aspects of the life sciences. In this paper the authors develop rEstus2, a powerful iterative step forward that we think certainly warrants publication in Nature Communications. Using rEstus the authors expand on their previously published findings to demonstrate bioelectric contact inhibition (BCI) which reduces bioelectric volatility as collective size increases. They molecularly characterize BCI and attribute it to gap junctions, but find that BCI does not affect calcium dynamics. They then integrate these data into a descriptive model. This is a very nice paper, and there are only a couple of comments:

Response: We thank the reviewer for the careful evaluation of our manuscript and for the very positive assessment of our work. We are particularly encouraged that the reviewer considers rEstus2s a meaningful advancement for the bioelectric toolkit and that the study is viewed as suitable for publication in *Nature Communications*. We also appreciate the thoughtful and constructive comments, which have helped us to further clarify and strengthen the manuscript.

-The strongest alternative technology for optically estimating membrane potential is the PeT dye system designed by Evan Miller's group. Recent work using Fluorescent Lifetime Imaging (FLIM) has greatly improved the utility of PeT dyes for estimating resting membrane potential (refs 1-4 below), but the authors do not mention the PeT system in the current version of this manuscript. Miller's PeT system has recently been used to demonstrate long-distance propagation of bioelectric but not calcium dynamic signals (currently in preprint, #5 below), which has interesting implications for this paper that probably should be discussed. A table comparing published relevant values for PeT dyes with rEstus1 and 2 and ASAP3 (as well as any others the authors find helpful) could be helpful to properly contextualize rEstus2.

Response: We thank the reviewer for this comment. We agree that PeT dyes represent a powerful alternative for voltage imaging, particularly in systems where genetic engineering is challenging. While rEstus2s offers superior voltage sensitivity, PeT dyes provide a complementary approach with distinct advantages e.g., for Fluorescence Lifetime Imaging Microscopy (FLIM).

As noted by the reviewer, recent work has greatly improved the utility of PeT dyes for estimating absolute V_m through FLIM. Interestingly, we have recently demonstrated that ASAP-type GEVIs are also suitable for absolute V_m quantification using FLIM, showing lifetime changes that are comparable to, or even exceed, those of PeT-based systems. This recent study (Nair *et al.*, 2025) addresses some of the challenges mentioned by the reviewer (preprint #5), such as dye internalization and self-quenching, which can complicate long-term measurements.

Concerning the table, we already have a table (Supplementary Table 1) that contains the relevant parameters for ASAP3, JEDI-1P, rEstus, and the sensors created in this paper. We included JEDI-1P because it belongs to the most recently developed sensors for one-photon voltage imaging. This includes absolute brightness values, which we were able to measure using mKate2s as a reference.

For the dyes, it is challenging to have a direct comparison. The only property directly comparable would be ΔF/F, which is typically given as the intensity change for a 100-mV step. As mentioned in the text, dyes and GEVIs have complementary use-case scenarios, and a direct comparison may not be feasible. However, we provide an example for ΔF/F of the best PeT dye we could find in terms of voltage sensitivity (dsVF2.2(OMe).Cl, ΔF/F 1.63-fold) compared to rEstus2s (ΔF/F 4.6-fold).

We added the following paragraph in the discussion and included the suggested references:

An alternative to GEVI-based approaches for bioelectric voltage imaging is the use of synthetic dye-based systems. In particular, PeT (photoinduced electron transfer)-based dyes have recently emerged as powerful tools in bioelectric research, as they can be calibrated for absolute V_m measurements using fluorescence lifetime imaging microscopy (FLIM) under appropriate conditions.[53–58] While these dyes generally exhibit lower voltage sensitivity (e.g., dsVF2.2(OMe).Cl shows a 1.63-fold intensity change over 100 mV)[59] than the most recent GEVIs, such as rEstus2s (4.6-fold change over 100 mV), they provide a valuable, non-genetic

alternative for tissue-level V_m imaging, particularly in contexts where genetic targeting is not feasible.

-rEstus2 is a powerful addition to the bioelectric toolkit, but its lack of ratiometry and lower brightness should be discussed in context of rEstus1. This is not a problem, and the sensitivity improvements of rEstus2 warrant publication in Nature Communications. But it might be good for readers to more clearly contextualizing rEstus2 as an alternative tool to rEstus1 instead of a clear 100% upgrade, to help readers decide on the best tool for their application.

Response: We thank the reviewer for this comment and agree that rEstus2s does not represent a universal upgrade over rEstus in all aspects. We have therefore revised the manuscript to more clearly delineate the respective use-case scenarios of both indicators. As noted in the manuscript, rEstus2s provides increased sensitivity but lacks the secondary excitation peak required for ratiometric measurements and is slightly dimmer than rEstus.

To better contextualize these trade-offs, we have added a sentence emphasizing that rEstus remains advantageous in specific applications.

Consequently, although rEstus is less sensitive than rEstus2s, it remains the preferred choice for absolute V_m calibration.

-The authors effectively demonstrate the Bioelectric Contact Inhibition (BCI) phenomenon and convincing implicate Connexin activity in its function. Their model (Figure 6a-6b) that gap junctions facilitate a formation of a functional syncytium is not supported by the lack of synchrony in calcium dynamics. Their explanation for this discrepancy needs more support with referenced literature. Many gap junctions are voltage gated, so would it be possible for example that fast bioelectric signals are able to pass through gap junctions before calcium waves are able to propagate? In any case, the contradictory calcium data should be clarified as a knowledge gap that must be addressed in future work.

Response: We agree with the reviewer that the lack of Ca^{2+} equilibration between neighboring cells appears counterintuitive in the context of gap junction-mediated coupling. Indeed, we initially expected at least partial synchronization of Ca^{2+} dynamics between adjacent cells. However, this was not observed in our experiments.

At the same time, our data using Cx43/Cx45 double-knockout cells demonstrate that V_m synchronization is abolished in the absence of gap junctions, supporting the conclusion that electrical coupling is mediated by connexins. We therefore interpret the apparent discrepancy between electrical coupling and Ca^{2+} dynamics as a consequence of fundamentally different transport mechanisms.

Specifically, electrical coupling is mediated by ionic currents that can equilibrate membrane potential across a coupled network on near-instantaneous timescales, whereas Ca^{2+} signaling depends on diffusion-limited processes that are inherently slower and more spatially restricted. In addition, intracellular Ca^{2+} is strongly buffered and often confined to micro- or nanodomains, which may further limit effective intercellular equilibration.

We have added a paragraph to the Discussion to clarify this distinction and to better support our interpretation.

Although connexins can indirectly support Ca^{2+} wave propagation through diffusion of signaling molecules such as IP_3,[23] the extent to which they permit direct Ca^{2+} flux remains unclear.[65] A functional separation between Ca^{2+} and V_m signaling has also been reported in neural crest explants.[57] The discrepancy between the observed functional electrical syncytium and the absence of coordinated Ca^{2+} dynamics remains an open question that warrants further investigation. A plausible explanation is that these processes are governed by fundamentally different biophysical constraints. Electrical coupling is mediated by ionic currents that rapidly equilibrate V_m across the network's shared capacitance nearly instantaneously. In contrast, Ca^{2+} flux through gap junctions is limited by diffusion through narrow pores and is further constrained by strong intracellular buffering. Consequently, the level of junctional conductance required for effective electrical coupling is likely substantially lower than that required to achieve intercellular equilibration of Ca^{2+}.

We appreciate the reviewer's suggestion regarding voltage-dependent gating of connexins. However, we consider this unlikely to play a major role under our experimental conditions. The spontaneous V_m fluctuations observed in our system are typically <10 mV, which is well below the transjunctional voltage differences (on the order of 20-50 mV) generally required to substantially modulate the conductance of connexins such as Cx43 and Cx45.

Finally, we agree that the coexistence of strong electrical coupling and limited Ca^{2+} synchronization highlights an important mechanistic gap. We have revised the manuscript to clarify that we have a "functional electrical syncytium" and that the functional role of electrical coupling requires further investigation in more physiologically relevant cell models.

However, the functional role of electrical coupling and signaling in non-excitable cells remains incompletely understood and requires further investigation in more physiologically relevant models, such as organoids derived from induced pluripotent stem cells.

-The idea of cell couples as an intermediate between individual and confluent cultures is quite clever, and a useful datapoint, but their assertion starting line 2 that "If suppression of Vm fluctuations in confluent cultures were primarily driven by physical cell-cell contact and activation of membrane surface receptor pathways, a substantial reduction in Vm volatility would be expected upon formation of cell pairs." is questionable without extensive literature support. A confluent cell layer is highly likely to be very different from a single or couple of cells in many critical ways, and it would be good to contextualize their claim with that in mind.

Response: We agree with the reviewer that a confluent cell layer represents a complex environment that differs qualitatively from cell pairs in several important aspects, including mechanical constraints and receptor signaling. Our statement was intended to distinguish between receptor-mediated signaling mechanisms and passive electrical effects arising from intercellular coupling.

If V_m stabilization was primarily driven by contact-dependent receptor activation, one might expect a pronounced reduction in volatility upon formation of the first stable cell-cell junction (i.e., in cell pairs). However, our data instead show that V_m variance decreases progressively with increasing network size, following an approximate 1/n scaling relationship. This gradual reduction is more consistent with a collective, biophysical mechanism than with a discrete signaling transition.

We fully agree that this observation alone does not establish equivalence between cell pairs and confluent monolayers with respect to receptor signaling. However, we further show that in the Cx43/Cx45 double-knockout model, V_m stabilization is abolished even in fully confluent cultures, where physical contact and receptor engagement are preserved. This indicates that cell-cell contact per se is insufficient to account for the observed effect and supports a primary role for gap junction-mediated electrical coupling in governing V_m stability across the network.

We have revised the text as follows:

If suppression of V_m fluctuations in confluent cultures were primarily driven by physical cell-cell contact and activation of membrane surface receptor pathways, one might expect a pronounced reduction in volatility upon formation of the first stable cell-cell junction.

-The authors discuss the interactions between membrane potential and Erk signaling document dynamic bioelectric signals regulated by collectivity. There is a growing body of literature that the Erk pathway temporally encodes information (see refs 6,7 below) and it would improve the impact of this manuscript were the authors to comment on the implications of the dynamic bioelectric signals they describe on the temporal dynamics of Erk signaling.

Response: Thank you for highlighting these relevant studies and for the suggestion to further discuss the connection to ERK dynamics.

We agree that, in addition to Ca^{2+} signaling, the ERK pathway is one of the most plausible downstream effectors of V_m -mediated bioelectric signals. Indeed, previous work has directly linked changes in membrane potential to enhanced Ras clustering and subsequent ERK activation. However, it remains an open question whether V_m exerts its effects primarily through steady-state shifts or through dynamic, time-varying signals. Importantly, ERK signaling itself is known to encode information in its temporal dynamics, where transient versus sustained activation can lead to distinct outcomes such as proliferation or differentiation. Moreover, stochastic ERK activity pulses have been shown to depend on cell density, further underscoring the role of collective cellular context in shaping signaling dynamics.

We have now added a paragraph to the manuscript:

A fundamental open question is whether V_m is instructive and, if so, whether the information is predominantly encoded as sustained steady-state changes or as transient fluctuations. It is well established that signaling outcomes can be encoded in the temporal dynamics of pathways such as ERK.[71] For example, transient ERK activation in response to EGF promotes proliferation, whereas sustained ERK activation drives differentiation.[72] Furthermore, stochastic ERK activity pulses have been shown to regulate proliferation rates in a cell density-dependent manner.[73] Thus, if ERK signaling is modulated by V_m, as suggested by previous studies,[6,7] its dynamics may likewise be shaped by the collective electrical properties of the multicellular network.

-In Figure 2H, could the difference in spectral density differences be attributed to capacitive current differences arising from the differential voltage sensitivity of the sensors?

Response: Thank you for this question. We agree that, in principle, differences in gating currents could contribute to changes in spectral density. However, in our case this is unlikely. The mutations introduced in rEstus2s do not alter the number of gating charges; instead, the improved voltage sensitivity arises primarily from modifications in the chromophore environment. These changes are not expected to significantly affect the magnitude of gating currents or introduce additional capacitive artifacts.

The observed 2.4-fold increase in spectral density is therefore best explained by the enhanced optical response (dF/F) of the sensor. This is consistent with the predicted improvement in sensitivity at the typical resting membrane potential of HEK293T cells (≈ -50 mV), where a ~1.55-fold increase in dF/F (Supplementary Figure S2) translates into an expected 2.4-fold (1.55^2) increase in spectral density. The experimental result is thus in good agreement with the theoretical prediction based on sensor performance.

We congratulate the authors on a very nice contribution to the field.

Response: We sincerely thank the reviewer for this encouraging comment. We greatly appreciate the positive assessment of our work and are pleased that it is viewed as a valuable contribution to the field.

References cited:

1 Lazzari-Dean, J. R., Gest, A. M. M. & Miller, E. W. Optical estimation of absolute membrane potential using fluorescence lifetime imaging. eLife 8, e44522 (2019). https://doi.org:10.7554/eLife.44522 ✓
2 Lazzari-Dean, J. R. & Miller, E. W. Optical Estimation of Absolute Membrane Potential Using One- and Two-Photon Fluorescence Lifetime Imaging Microscopy. Bioelectricity 3, 197-203 (2021). https://doi.org:10.1089/bioe.2021.0007 ✓
3 Gest, A. M. M., Grenier, V. & Miller, E. W. Optical Estimation of Membrane Potential Values Using Fluorescence Lifetime Imaging Microscopy and Hybrid Chemical-Genetic Voltage Indicators. Bioelectricity 6, 34-41 (2024). https://doi.org:10.1089/bioe.2023.0027 ✓
4 Gest, A. M. M. et al. A red-emitting carborhodamine for monitoring and measuring membrane potential. Proc Natl Acad Sci U S A 121, e2315264121 (2024). https://doi.org:10.1073/pnas.2315264121 ✓
5 McMillen, P. & Levin, M. The neural crest as a bioelectric Rosetta Stone: translating the analog and digital bioelectric code with Fluorescent Lifetime Imaging (FLIM). . OSF Preprints (2025). https://doi.org:10.31219/osf.io/ncx84_v1 ✓
6 Purvis, J. E. & Lahav, G. Encoding and decoding cellular information through signaling dynamics. Cell 152, 945-956 (2013). https://doi.org:10.1016/j.cell.2013.02.005✓
7 Goglia, A. G. et al. A Live-Cell Screen for Altered Erk Dynamics Reveals Principles of Proliferative Control. Cell Syst 10, 240-253 e246 (2020). https://doi.org:10.1016/j.cels.2020.02.005✓

Reviewer #3 (Remarks to the Author):

I co-reviewed this manuscript with one of the reviewers who provided the listed reports. This is part of the Nature Communications initiative to facilitate training in peer review and to provide appropriate recognition for Early Career Researchers who co-review manuscripts.

Reviewer #4 (Remarks to the Author):

I co-reviewed this manuscript with one of the reviewers who provided the listed reports. This is part of the Nature Communications initiative to facilitate training in peer review and to provide appropriate recognition for Early Career Researchers who co-review manuscripts.

REVIEWER COMMENTS

Changes to the text of the manuscript are highlighted in cyan

The updated figures are attached as PDF files.

All source data, including the exact *p*-values and the statistical tests used, are attached as a source/supplementary data files.

After reviewing all statistical analyses, we made the following corrections:

Figure 3e (top)

After excluding duplicate cell pairs and considering each cell pair only once, the new *p*-value is 0.11 and is therefore not statistically significant.

Figure 3h (Individual)

The comparison between Ctrl and ANO1 is now indicated with a single asterisk (*) (p = 0.02).

Figure 3i

Similar to the ANO1 and KCa3.1 groups, the difference between individual and confluent Ctrl cells is also not statistically significant (p = 0.29).

Figure 5d (HEK293T)

After excluding duplicate cell pairs and considering each cell pair only once, the new *p*-value is 0.07 and is therefore not statistically significant.

Reviewer #1 (Remarks to the Author):

The authors have now addressed all my minor comments for improvement.

Thus, I should recommend the revised manuscript in its present form.

Response: We thank the reviewer for their valuable comments and for taking the time to help us improve our manuscript.

Reviewer #2 (Remarks to the Author):

I think the authors did a really nice job responding to all the comments, and the manuscript is much improved. I think it can be published now, I have no more concerns.

Response: We thank the reviewer for the positive assessment and for their time in reviewing our paper.

My co-reviewer said:

> My one comment is that I would like to see an addition to the paragraph at the end of their page 10 of the response document noting that ratiometry or FLIM are key in addressing artifacts stemming from cell shape change or differential sensor expression/staining.

Response: As requested, we have added the following sentence to the indicated paragraph to address this:

Ratiometric and FLIM-based approaches are superior in situations where motion artifacts, dye distribution variability, or differences in GEVI expression levels need to be accounted for.
